# AS7341 Spectral Sensor with Machine Learning for Non-Contact Temperature Monitoring in Electrolytic-Plasma Hardening

**DOI:** 10.3390/s26103080

**Published:** 2026-05-13

**Authors:** Rinat Kussainov, Aikyn Erboluly, Zhanel Bakyt, Nurlat Kadyrbolat, Rinat Kurmangaliyev, Bauyrzhan Rakhadilov, Vladislav Koc, Aknur Rakhmetollayeva, Zarina Satbayeva

**Affiliations:** 1 Engineering Center “Strengthening Technologies and Coatings”, Shakarim University, Semey 071412, Kazakhstan; aikynerboluly0@gmail.com (A.E.); bakytzhanel@gmail.com (Z.B.); ersinnur44@gmail.com (N.K.); rinat_real@rambler.ru (R.K.); kotsvladislav1@gmail.com (V.K.); rachmettolaeva@icloud.com (A.R.); 2 Department of Technical Physics and Heat Power Engineering, Research School of Physical and Chemical Sciences, Shakarim University, Semey 071412, Kazakhstan; rakhadilovb@mail.ru (B.R.); satbaeva.z@mail.ru (Z.S.); 3 Plasma Science LLP, Ust-Kamenogorsk 070000, Kazakhstan

**Keywords:** AS7341, electrolytic-plasma hardening, non-contact temperature measurement, feature selection, machine learning, regression models, NIR/Clear, metallographic analysis, microhardness

## Abstract

Electrolytic-plasma hardening of steel components requires reliable non-contact temperature monitoring, but traditional pyrometry is complicated by the variable emissivity of steel and the intense radiation of the plasma envelope. This work presents an approach that repurposes a compact multispectral AS7341 sensor into a virtual temperature sensor based on physically grounded spectral feature engineering and regularized machine learning. The use of logarithmic ratios of the near-infrared channel (940 nm) to the visible channels suppresses the plasma contribution and linearizes Wien’s radiation law. On a controlled dataset of 20 cycles, this increases the Pearson correlation with the peak temperature from r = 0.498 (raw NIR channel) to r = 0.781 for the log(NIR/Clear) feature. Current is identified as a confounding variable; normalizing the NIR/Clear ratio by the cycle-averaged current (r = 0.761) ensures correct signal interpretation under varying process conditions. Two narrow channels–NIR (940 nm) and F8 (680 nm)–provide accuracy equivalent to the broadband Clear channel (r = 0.778 vs. 0.781), thus simplifying hardware implementation. Ridge regression using three weakly correlated features (log(NIR/Clear), cycle duration, and initial temperature) achieves a mean absolute error of 91.4 °C under leave-one-out cross-validation (LOOCV) and 85.5 °C on an independent current-group test (R^2^ = 0.536). Independent verification by scanning electron microscopy and Vickers microhardness on 30KhGSA steel confirms reliable separation of the three thermal regimes: underheating (<800 °C, 280–320 HV), optimal quenching (800–900 °C, 620–680 HV, fine-needle martensite), and overheating (>900 °C, 540–590 HV). The proposed set of spectral features provides a physically justified basis for a low-cost industrial temperature sensor for electrolytic-plasma processing.

## 1. Introduction

Electrolytic-plasma hardening (EPH) is currently considered one of the most promising technologies for modifying the surface of structural and tool steels. This method provides a unique combination of extremely high heating rates (20–500 °C/s) and cooling in thin surface layers, which allows for significant improvements in performance characteristics while maintaining the ductile core of the material [1,2]. The physicochemical uniqueness of the process lies in the formation of a stable vapor-gas shell (VGS) around the workpiece, acting as a plasma electrolyzer with critical heat flux densities of up to 10^7^–10^8^ W/m^2^. The practical efficiency of EPH has been confirmed for various industries: when used for woodworking tools (65G, 9HS steel), edge wear resistance increases by 2.5–3 times; for oil and gas valve parts, erosive wear is significantly reduced; for gears and crankshafts of agricultural machinery, contact fatigue increases by 30–50% while maintaining geometric accuracy. The work of B.K. Rakhadilov et al. describes achieving a hardness of over 900 HV with strict adherence to the temperature regime. The study by S.A. Kusmanov et al. showed that combined treatment—anodic plasma-electrolytic saturation followed by quenching and plasma-electrolytic polishing—can increase the wear resistance of steel by 21.5 times (after boriding) and by 1.8 times (after nitrocarburising) compared to untreated samples [3,4]. The works of Yu.N. Tyurin and A.D. Pogrebnyak demonstrated that ultra-high heating rates shift the austenitic transformation to higher temperatures, enabling the production of supersaturated solid solutions and finely dispersed structures unattainable with traditional bulk quenching [2]. Research by M.K. Skakov and B.K. Rakhadilov confirmed the formation of a hardened layer with a gradient structure: from near-surface martensite to troostite–martensite and the original ferrite–pearlite structure [5]. The work of P.N. Belkin revealed the mechanism of “electrolyte acceleration” of diffusion, enabling carburizing and nitriding in just 2–5 min [6].

Nevertheless, the widespread adoption of EPH is limited by the critical problem of reproducibility of results. The physical complexity of the electrolytic-plasma process is highlighted by recent studies of discharge types during cathodic plasma electrolysis. Forschner et al. [7] combined optical emission spectroscopy, high-speed imaging, and electron microscopy to show that the plasma in the vapor layer is not a homogeneous glow discharge but consists of multiple filamentary discharges with arc-like and spark-like characteristics. They reported electron densities of 1.2–9.8 × 10^15^ cm^−3^ and cathode spot current densities up to 42 mA cm^−2^, while the discharge voltage remained in the range of 120–250 V, indicating the simultaneous existence of glow and arc modes. Based on the rotational spectra of the hydroxyl radical, the authors obtained two gas temperatures: approximately 1650 K and 6580 K, with the lower temperature (1650 K) considered more representative of the actual gas temperature. Dehghani [8] showed that electrode roughness affects the localization of microdischarges and the distribution of active species (OH, H_2_O_2_) at the plasma–liquid interface, thereby determining the morphology of the formed nanostructures. These data confirm that the reproducibility of spectral monitoring in EPH requires consideration not only of electrical parameters but also of the surface state of the sample. Additionally, Saifutdinov et al. [9] developed a self-consistent model of a glow discharge in argon with a liquid anode, taking into account electrode heating, water evaporation, and the kinetics of hydrated ions. Their study showed that the plasma-forming ion changes from Ar_2_^+^ to the hydrated cluster H_9_O_4_^+^, and that the negative charge is carried not only by electrons but also by OH^−^ ions, which emphasizes the complex ionic composition of the plasma in contact with a liquid. The enumerated complexities directly create a key barrier to the practical application of electrolytic-plasma hardening technology, including the possibility of precise and inertialess temperature control directly in the plasma discharge zone.

Existing methods based on standard pyrometers face substantial errors caused by two main factors: the dynamic instability of the emissivity coefficient ε due to the formation of oxide films of variable composition, and “optical noise” from the vapor-gas envelope, which screens the thermal radiation of the part and creates its own discrete spectrum [10]. A promising alternative to single-channel pyrometers is optical emission spectroscopy (OES): it analyzes the shape of the spectrum, allowing one to separate the “gray” continuum of thermal radiation from the metal and the discrete lines of plasma emitters in combination with machine learning. The work of Hussein et al. showed that spectral line analysis allows one to calculate plasma temperatures in the range of 6000–10,000 K [11]. A significant contribution to the validation of ML for plasma spectroscopic diagnostics was made by Samuell et al.: a neural network (12 hidden layers, 13,741 parameters) trained on 1865 EUV/VUV spectra from the DIII-D tokamak achieved MAE < 1 eV at Te < 10 eV, and the plasma state classifier showed F1 = 0.96 at a registration rate 10 times higher than the reference Thomson scattering method [12]. The authors’ key methodological conclusion is that spectrum normalization by maximum intensity (using relative line ratios) reduces the requirements for the dynamic range of the instrument from 10^8^ to 10^3^.

The transition from rigid analytical formulas to machine learning allows one to circumvent the limitations of traditional radiation physics in plasma conditions. The model does not rely on predetermined laws, but independently generates an algorithm for determining the temperature, learning from correlations between the discrete spectrum and verified data. This ensures flexible adaptation to nonlinear changes in the spectral composition caused by the influence of the electrolyte. The review by Trieschmann et al. [13] systematizes the application of machine learning in low-temperature plasma (LTP) physics, highlighting supervised learning as the most mature direction for diagnostic tasks. The authors point to a key advantage, which lies in the ability of ML methods to identify “unknown unknowns”—hidden patterns that analytical models are fundamentally unable to detect. At the same time, they emphasize that it is precisely this feature that opens up new prospects for the development of plasma science and plasma-based technologies.

As mentioned above, direct contact temperature measurement under EPH conditions is impossible due to the aggressive electrolyte environment and high-voltage potential. Traditional pyrometers are limited by the uncertainty of the emissivity and scattering by the vapor envelope. In this context, the use of the multichannel spectral sensor AS7341, which records integral signals in 10 spectral bands (415–940 nm) directly during heating, is promising. Unlike traditional pyrometers operating with one or two spectral channels, the AS7341 generates a 10-dimensional vector of spectral responses. As shown in a recent study by Malik and Ganotra [14], this compact sensor is successfully used to estimate the correlated color temperature (CCT) of displays, demonstrating high accuracy and cost-effectiveness, and its small size and portability open up possibilities for use in various spectroscopy applications. This confirms the potential of AS7341 for constructing nonparametric models of the spectrum-temperature relationship that compensate for the uncertainty associated with the variability of the plasma sheath.

Two-color (spectral ratio) pyrometry is widely used to solve the problem of contactless temperature measurement under plasma conditions. As noted in the work of Voronin et al. [15], this approach can significantly reduce the methodological error associated with the unknown emissivity of an object, since the ratio of signals at two closely spaced wavelengths depends primarily on temperature. The authors showed that at plasma temperatures above 10 eV and moderate particle concentrations, the near-wall plasma can be considered transparent to thermal radiation in the 3–4 µm range, substantiating the applicability of two-color pyrometry under plasma conditions.

In metallurgy, machine learning methods have already proven themselves useful for predicting the thermal state of liquid steel. For example, Shtangret et al. [16] developed models based on the FEM solution of the Fourier equation, as well as decision trees, linear regression, and neural networks, to predict the cooling rate of steel in a ladle. The authors demonstrated that even when working with noisy production data, neural network models achieve a determination coefficient of R^2^ = 0.78, supporting their use in real-time decision support systems. These results confirm the feasibility of applying machine learning methods to thermal control of metallurgical processes and inspire a similar approach for temperature control during EPH.

However, the widespread use of ML in materials science and related fields faces a fundamental limitation: available experimental datasets often contain only tens or hundreds of observations, which is significantly smaller than those typical for other fields of science [17]. As shown in [18,19], it is the small sample size, rather than the complexity of the algorithm, that is the key factor limiting the predictive power of ML models. However, the limited size of the data itself is not an insurmountable obstacle: there are developed strategies for extracting consistent patterns from limited samples. These include careful feature engineering [20], the use of regularized models robust to overfitting [21], and the application of specialized validation schemes such as leave-one-out cross-validation (LOOCV), which provides an unbiased quality estimate for small sample sizes *n* [22,23]. In this paper, we follow precisely this methodology, focusing not on building a “deep” model, but on identifying physically based features and assessing the fundamental applicability of the AS7341 spectral sensor for indirect temperature monitoring during the EPH process.

This paper proposes an approach based on the AS7341 multichannel spectral sensor, which implements the principle of multiwavelength pyrometry—“spectral fingerprinting”—of the EPH process. The two-color pyrometry methodology is adapted to this sensor through the use of spectral channel ratios in the near-infrared range, specifically the NIR/Clear ratio and derived features, which combine the compactness of the sensor with the information content of spectral analysis. Unlike single-channel pyrometers, temperature is estimated here not by absolute radiation intensity, but by the shape of the spectral distribution, which allows for separation of the “gray” continuum of the heated metal and the discrete emission lines of sodium plasma (the doublet of the D-lines of Na ~589 nm). This minimizes the influence of variable emissivity and background glow of the electrolyte. The analysis was conducted on a controlled sample of 20 cycles with a fixed heating current; the NIR/Clear ratio, a spectrally invariant indicator of plasma color temperature, was used as the key feature. This study, for the first time, systematically selected features for machine learning tasks and assessed their significance in linear and ensemble models.

## 2. Materials and Methods

### 2.1. Spectral Instrumentation

**Figure 1:**
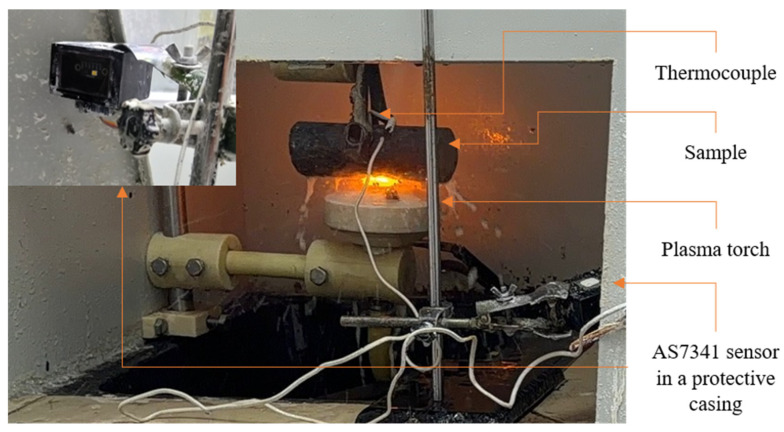
The EPH installation during the measurement of spectral and temperature values.

A multi-channel spectral sensor AS7341 (AMS-OSRAM, Premstaetten, Austria) was used, recording integral signals in 10 spectral bands: F1 (415 nm), F2 (445 nm), F3 (480 nm), F4 (515 nm), F5 (555 nm), F6 (590 nm), F7 (630 nm), F8 (680 nm), NIR (940 nm), and the broadband Clear channel. The sensor was placed in the EPH setup at a distance of 350 mm from the sample surface where heating took place (Figure 1). Signal integration was performed over the entire heating cycle, which allowed us to obtain integral radiation characteristics. The selection of informative spectral channels for the AS7341 sensor was based on the results of a spectroscopic study of an electrolyte-plasma discharge during hardening of 20GL steel, conducted by the authors using a high-resolution spectrometer in the 200–1000 nm range [24,25]. The discharge spectrum contains several characteristic groups of lines, each of which carries physically significant information about the plasma state. The iron lines Fe I and Fe II are distributed across the entire spectral range and reflect the composition of the steel being processed; the manganese line Mn I (~403 nm) corresponds to the alloying element of the alloy. Hydrogen lines Hβ (486.28 nm) and Hα (656.24 nm) are due to the dissociation of electrolyte water in the plasma discharge. The most intense components of the spectrum are the sodium Na I lines: the D-doublet at 589.0/589.6 nm (channel F5 of the AS7341 sensor), the 568.87 nm line (channel F5), the 616.25 nm line (channel F6), and the 819.5 nm line (NIR channel). The intensity of the sodium D-lines reached the detector saturation limit (65,535 relative units), which is explained by the exceptionally high emission activity of Na during the cathodic discharge.

The sample temperature was measured using a K-type thermocouple (wire diameter 0.2 mm). The thermocouple junction was spot-welded to the inner side of the sample (directly opposite the heating zone) through a through-hole drilled at a distance of 1 mm from the surface. This ensured direct contact with the base metal. A microcontroller controlled and collected data, configuring the sensor, conducting cyclic polling at a specified frequency, receiving thermocouple data, and synchronously recording all channels with timestamps in CSV format. Software written in PureBasic (v6.21) ensured synchronous reception and recording in real time. Each CSV line contained a timestamp, the values of channels F1–F8, NIR, and Clear, and the current thermocouple temperature. The recording frequency was ≈5.5 Hz.

Prior to the experiments, the AS7341 sensor was calibrated in an SNOL 8.2/1100 laboratory furnace (UAB “SNOL”, Panevėžys, Lithuania). At each temperature level (from 50 °C to 1000 °C in 1 °C steps), 1000 consecutive readings were recorded to ensure statistical reliability. The sensor operated with the following fixed settings: ATIME = 31 (integration time ≈ 182 ms), ASTEP = 599, and GAIN = 64×. The raw 16-bit ADC counts (Raw_Counts) were logged without conversion to Basic Counts, because with constant gain and integration time, such conversion does not affect correlation relationships. Dark-current subtraction (offset) and NIR correction were not applied, since the features used in this work are signal ratios—they automatically compensate for constant additive offsets, and NIR crosstalk under plasma discharge conditions is negligible compared to the useful signal. The calibration data were used only to verify the linearity of the sensor; the final model was built on signal ratios, making it robust against absolute intensity variations.

### 2.2. Sample Description and Cycle Definition

From the total experimental dataset for electrolytic plasma heating, 20 heating cycles were selected in which the average current remained constant within each group: 60 A at 280 and 300 V, and 50 A at 320 V. This allows the AS7341 spectral signals to be considered as a function of temperature and heating duration, rather than current. In the context of EPH, a cycle represents a complete heat treatment process, including discharge initialization by applying voltage and plasma sheath formation, an active heating phase lasting duration_s, reaching the thermal peak (t_max_sensor), and cooling. Each observation in the sample corresponds to one cycle. The sample parameters are presented in Table 1.

**Figure 2:**
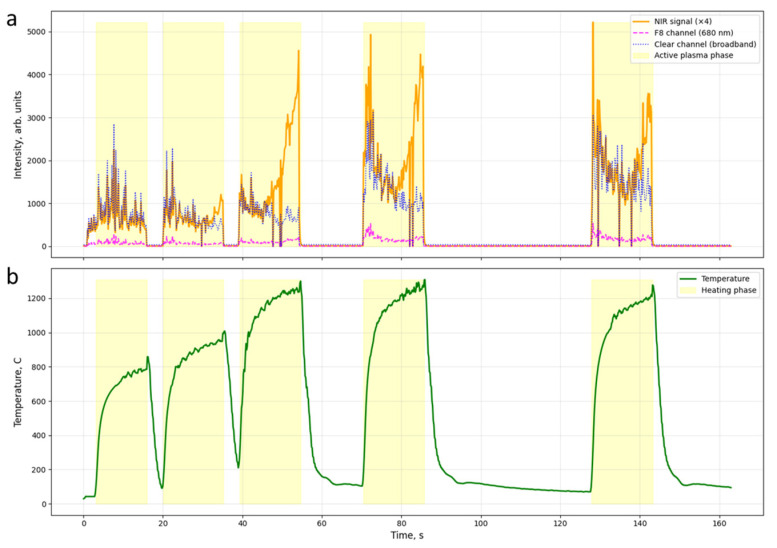
Examples of thermal cycles of the EPH and spectral signals of AS7341: (**a**) time traces of the NIR channel (orange, ×4 scaling), F8 channel (magenta), and Clear channel (blue); (**b**) corresponding thermocouple temperature curve (green).

For a visual representation of the cycle detection process and the relationship of spectral signals with temperature, Figure 2 shows a time trace for several processing cycles. The orange line (NIR × 4) in Figure 2a represents the signal of the NIR channel (940 nm), scaled for clarity; the magenta line corresponds to the F8 channel (680 nm, dark red region); and the blue line corresponds to the Clear channel. The green curve (Figure 2b) corresponds to the temperature recorded by the thermocouple. The yellow shading indicates the phase of active plasma combustion, detected by the NIR signal exceeding a threshold level.

Analysis of the time profile allows us to distinguish five successive phases. During the discharge initialization phase (t ≈ 1–2 s), a sharp jump in the NIR signal above the 100-unit threshold is observed, marking the formation of the plasma sheath; simultaneously, the surface temperature begins to rise. This is followed by the active heating phase (t ≈ 2–11 s), characterized by stable plasma combustion, during which the NIR signal remains steadily above the 60-unit threshold, and the temperature increases monotonically to a peak value. During the plasma decay phase, the NIR signal drops below the 60-unit threshold, marking the end of the pulse; however, the temperature continues to rise due to the thermal inertia of the steel. The thermal peak is reached within 1–2 s after the end of the pulse, which is explained by the propagation of heat from the surface into the depth of the part. At the final stage of cooling, the temperature decreases monotonically due to heat transfer into the mass of the part and into the electrolyte.

### 2.3. Feature Space for Machine Learning

Each observation contains initial variables that form the basis for constructing the feature space. The initial features include: peak surface temperature of the part t_max_sensor (target variable, °C), heating cycle duration_s (s), initial temperature of the part before the cycle t_start (°C), average voltage avg_volt (V) and current avg_curr (A), as well as integrals of the signals of 10 AS7341 channels per cycle (integral_f1–integral_f8, integral_clear, integral_nir).

To expand the informativeness of the model and take into account the physical laws of thermal radiation, derived features (feature engineering) were generated. Their generation is based on the assumption that it is the qualitative feature space, rather than the complexity of the model, that determines success when working with small samples [18]. The following groups of features were formed:

− Channel ratios (F_i/F_j, i > j)–normalization to absolute intensity, reducing the influence of distance to the part and current density variations.
− Spectral-temporal (F_i × duration)–accounting for the accumulated “light dose” over the heating cycle.
− Key ratio (NIR/Clear)–analogous to two-color pyrometry, characterizing the color temperature of plasma radiation.
− Logarithmic (log(NIR/Clear))–linearization of the exponential dependence of radiation intensity on temperature (according to Planck’s law).
− Quadratic ((NIR/Clear)^2^)–accounting for possible nonlinear effects.

The total number of generated features (including the original spectral channel integrals and their derivatives) was 62. This number includes direct integrals of 10 AS7341 spectral channels, thermal characteristics, and derived features: channel ratios, their logarithmic and quadratic transformations, combinations with cycle duration, and normalization to current.

### 2.4. Machine Learning Methodology for Small Samples

In conditions of limited data volume (*n* = 20), the choice of ML methodology is critical for obtaining reliable results. We relied on approaches proven effective in working with small samples in materials science and engineering applications [17,18,19,20,21,22,23]. The procedure for selecting features and constructing regression models included several sequential stages.

The key emphasis in the work is on the construction of physically based features (feature engineering), rather than on model complexity. Instead of using “deep” architectures that require large amounts of data, the main focus is on the formation of a high-quality feature space, which, as shown in the literature [17], determines success for small *n*. For this purpose, derived features were generated, including spectral channel ratios, their logarithmic and quadratic transformations, and combinations with cycle duration. To construct the regression dependencies, Ridge regression (L2 regularization) and Random Forest (ensemble method) were used, which by their nature are less prone to overfitting on small samples compared to non-regularized linear models or deep neural networks [16,21,26,27]. Quality assessment was performed using Leave-One-Out Cross-Validation (LOOCV), where each iteration trains the model on n-1 samples and tests on the remaining one. This method yields a virtually unbiased error estimate and is standard for small samples [21,23,26], in contrast to the standard split into training and test samples, which, for small *n*, leads to high estimate variance. To further evaluate the generalization ability of the model under different process conditions (50 A and 60 A currents), an independent validation was performed using grouped data splitting (GroupKFold). The model was trained on all cycles with one current value and tested on cycles with the other current.

### 2.5. Metallographic Analysis and Microhardness Measurement

The studied material is structural alloy steel 30KhGSA (analog of AISI 4140). Its chemical composition is given in Table 2 [28].

**Figure 3:**
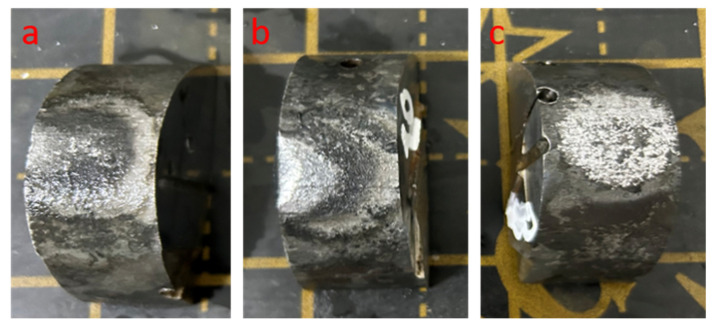
30KhGSA steel samples after electrolytic-plasma hardening: (**a**) optimal hardening (800–900 °C); (**b**) overheating (>900 °C); (**c**) underheating (<800 °C).

The working electrolyte was an aqueous solution of sodium carbonate Na_2_CO_3_ with a concentration of 20% by weight. This solution was chosen due to its high electrical conductivity, compositional stability, and characteristic plasma spectrum with a pronounced sodium doublet (~589 nm), suitable for spectral monitoring. Sections were prepared for analysis using a standard method: sequential mechanical grinding and polishing followed by final chemical etching in a 4% HNO_3_ solution in ethyl alcohol, which ensures the contrasting appearance of grain boundaries and phase boundaries. The appearance of 30KhGSA steel samples after electrolytic-plasma hardening is shown in Figure 3, where traces of local plasma heating on the surface are visible.

Microstructural studies of 30KhGSA steel samples subjected to EPH were carried out using scanning electron microscopy on a JSM-6390LV JEOL instrument (JEOL Ltd., Tokyo, Japan). Quantitative assessment of the mechanical properties of the surface layer was performed using the Vickers microhardness measurement method on an HLV-1 DT setup (Shanghai Hualong Test Instruments Corporation, Shanghai, China). To construct the hardness profile, indentation was performed over a grid of points on the cross-section of the sample—from the surface to the depth—which made it possible to determine the thickness of the hardened layer and the nature of the hardness gradient [29,30]. Each value represents the average of several independent measurements. The results of metallographic analysis were used to verify the operation of the ML classifier: the correspondence between the detected temperature regime and the observed structural composition serves as independent confirmation of the correctness of the developed monitoring system.

## 3. Results

### 3.1. Direct Channels of the AS7341 Sensor

The correlations of the integral signals of the direct AS7341 channels with the peak temperature for the controlled sample are presented in Table 3.

The direct AS7341 channels in their raw form show low informativeness. A statistically significant correlation with temperature (*p* < 0.05) is observed only for the NIR channel (r = 0.498). For the remaining channels (F1–F8, Clear), the correlation coefficients do not exceed 0.3, and the *p*-values are >0.05, indicating the absence of a significant linear relationship. This indicates that the absolute radiation intensity in individual spectral bands is not a reliable indicator of temperature due to the influence of factors such as the state of the plasma envelope (optical thickness, presence of discrete emission lines) and current density variations, while the geometric factor (distance to the part) remained constant (350 mm) and did not contribute to the observed variability. The results of the correlation analysis for the direct channels necessitate a transition to derived features (channel ratios, logarithmic transformations) to build reliable models.

### 3.2. Derived Features

To increase the informativeness of the spectral data, derived features were formed, including channel ratios, their logarithmic and quadratic transformations, as well as combinations with cycle duration. The correlations of the most significant derived features with the peak temperature are presented in Table 4.

**Figure 4:**
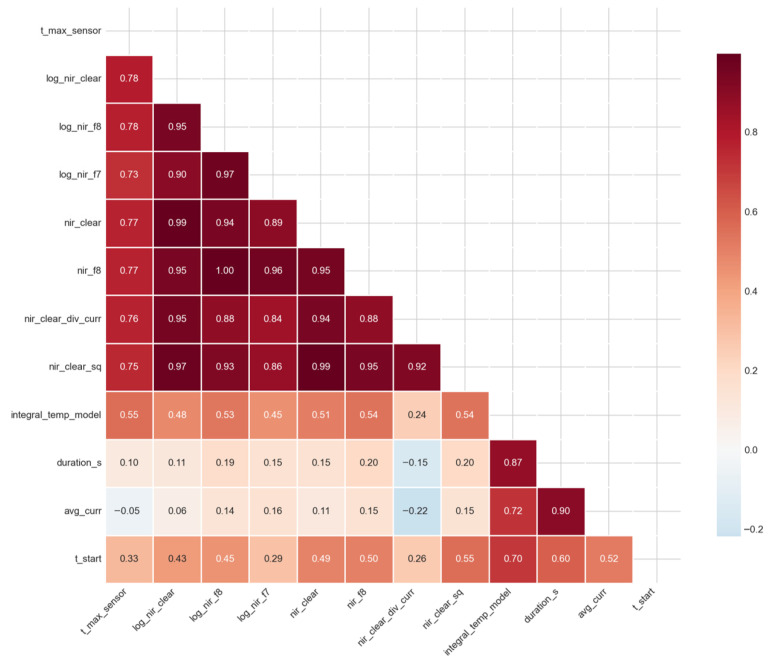
Correlation matrix of features.

To increase the informativeness of the spectral data, derived features were generated, including channel ratios, their logarithmic and quadratic transformations, as well as combinations with cycle duration. The correlations of the most significant derived features with the peak temperature are presented in Table 4, and Figure 4 shows the correlation heatmap of the key features.

**Figure 5:**
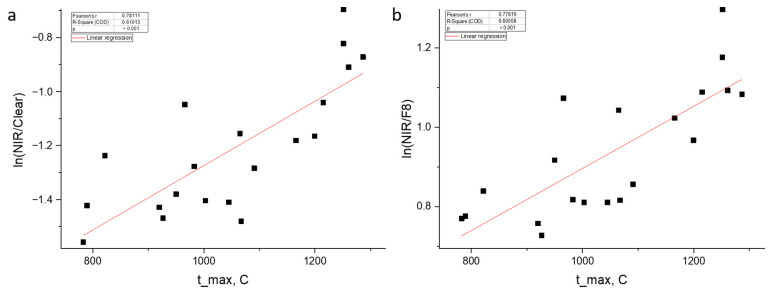
Dependence of spectral ratios on temperature: (**a**) log(NIR/Clear) vs. t_max_sensor; (**b**) log(NIR/F8) vs. t_max_sensor.

Figure 5 shows scatter plots for two key spectral features—log_nir_clear and log_nir_f8. The presented data allow several key conclusions to be drawn about the patterns linking the spectral characteristics of plasma emission with the steel surface temperature. The highest correlation coefficient (r = 0.781) is demonstrated by the log_nir_clear feature. As can be seen from the scatter plot (Figure 5a), the data points fall on a linear trend with minimal scatter. The direct ratio nir_clear (r = 0.769) gives a slightly lower correlation and a less linear dependence, confirming the need for logarithmic transformation.

The correlation of the log_nir_f8 feature with temperature, r = 0.778, is only slightly (by 0.003) lower than that of log_nir_clear. This is a fundamentally important result, demonstrating that two narrow spectral channels in the long-wave region (940 and 680 nm) may be sufficient for temperature estimation, without using the broadband Clear channel. The scatter plot for log_nir_f8 (Figure 5b) also shows a stable linear relationship comparable to that of log_nir_clear.

To assess the robustness of the spectral feature log_nir_f8, an analysis of its stability against synthetic Gaussian noise was performed. Noise with relative levels of 5%, 10%, 15%, and 20% of the standard deviation of the corresponding channel was added to the integral signals of the NIR (940 nm) and F8 (680 nm) channels. For each noise level, the correlation of the log(NIR/F8) feature with the temperature t_max_sensor was calculated. The results are presented in Table 5.

At noise levels up to 10%, the correlation remains high (r ≥ 0.73), indicating moderate robustness of the feature. At 20% noise, robustness drops sharply, highlighting the need for effective shielding of the AS7341 sensor from electromagnetic interference and stabilization of recording conditions in industrial environments.

The product of the spectral ratio and the cycle duration (nir_clear_x_dur) gives a correlation of r = 0.580, which is lower than that of the ratio itself without time consideration. This indicates that at a fixed current and a relatively narrow range of cycle durations (7.9–15.3 s), the direct logarithmic transformation contributes more to informativeness than accounting for the time scale. However, in ensemble models (Random Forest), the nir_clear_x_dur feature demonstrates the greatest importance, which will be discussed in detail later.

The quadratic transformation nir_clear_sq (r = 0.746) shows a result close to linear relationships, indicating the possibility of a non-linear dependence between the spectral ratio and temperature. This opens up prospects for using more complex regression models.

The transition from absolute values to channel ratios and their logarithmic transformations allows increasing the correlation coefficient with temperature from 0.498 (for the direct NIR channel) to 0.781 (for log_nir_clear). The obtained result confirms that the targeted formation of derived features is a necessary step in building reliable machine learning models for spectral thermometry tasks.

The correlation heatmap (Figure 4) demonstrates a high mutual correlation between log_nir_clear, nir_clear, nir_clear_sq, and nir_clear_div_curr (r > 0.9), which is explained by the mathematical relationship between these features. At the same time, duration_s and integral_temp_model show a high correlation (r = 0.87), which is expected since the integral of the model temperature directly depends on the heating time.

An interesting feature revealed during the analysis of a series of consecutive cycles is the relationship between the cycle number, the initial temperature of the sample, and the NIR/Clear spectral ratio. As the cycle number increases, a monotonic increase in the initial temperature t_start is observed, indicating the accumulation of heat by the part. This dynamics is accompanied by an increase in the peak temperature and a rise in the NIR/Clear ratio. This fact indicates that the spectral sensor records not only the instantaneous discharge parameters, but also the cumulative thermal effect accumulated by the sample during previous processing cycles.

Thus, the conducted analysis of derived features made it possible to identify the most informative spectral characteristics—logarithmic transformations of the NIR/Clear and NIR/F8 ratios. It can also be added that the nir_clear_div_curr feature (normalization by current) retains a high correlation (r = 0.761), confirming the need to account for current as a confounder. In a real EPH control system with variable current, this normalization becomes critically important for the correct interpretation of spectral signals.

**Figure 6:**
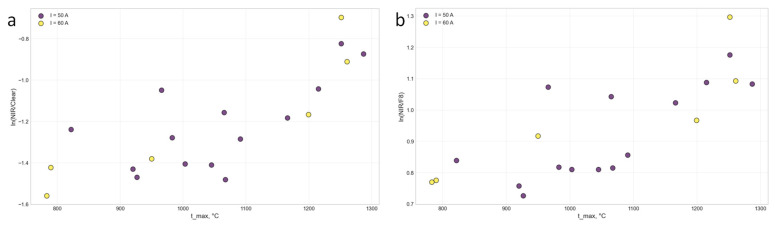
Dependence of (**a**) ln(NIR/Clear) and (**b**) ln(NIR/F8) on temperature (color–current).

Figure 6 shows the dependence of NIR/Clear on temperature with color coding by current.

It can be seen from the graphs that at the same temperatures, cycles with a current of 60 A (orange points) have higher NIR/Clear and NIR/F8 values. This confirms that current is a confounder: at a fixed temperature, a higher current produces a brighter plasma [31]. Therefore, in the full sample (without current fixation), correlations decrease, while in the controlled sample, they increase. This effect also explains why, in uncontrolled conditions, the direct AS7341 channels show a negative correlation with temperature: at a higher current (60 A), the plasma brightness is higher than at 50 A, even if the temperature is lower. The fixation of current in this study allowed this distorting factor to be eliminated and the true dependence of spectral ratios on temperature to be revealed.

### 3.3. Assessment of Feature Importance in ML Models

To identify the most informative spectral characteristics, feature importance analysis was performed using two types of models: Ridge regression, which allows assessing the stability of coefficients under L2 regularization, and Random Forest, which provides an estimate of importance based on Gini importance.

In the Ridge regression model, the highest normalized coefficient (1.00) was obtained for the log_nir_f8 feature, confirming the high informativeness of the logarithmic ratio of NIR (940 nm) to channel F8 (680 nm). Slightly inferior to it are nir_clear_div_curr (0.999) and log_nir_clear (0.913). High importance is also demonstrated by nir_f8 (0.881) and log_nir_f7 (0.877). The independent inclusion of the cycle duration duration_s and the initial temperature t_start makes a noticeably smaller contribution, indicating the dominance of spectral information over temporal parameters at a fixed current.

The Random Forest feature importance analysis revealed a different picture: the highest normalized importance (1.00) was obtained for the nir_clear_x_dur feature—the product of the NIR/Clear spectral ratio and the cycle duration, confirming the importance of accounting for accumulated heat input. The second most important is log_nir_f8 (0.771), which is consistent with the Ridge regression results. High importance is also demonstrated by nir_f8 (0.649), nir_clear_sq (0.481), and log_nir_f7 (0.441). The direct ratio nir_clear (0.347) occupies a lower position, indicating the need for logarithmic transformation to increase informativeness.

**Figure 7:**
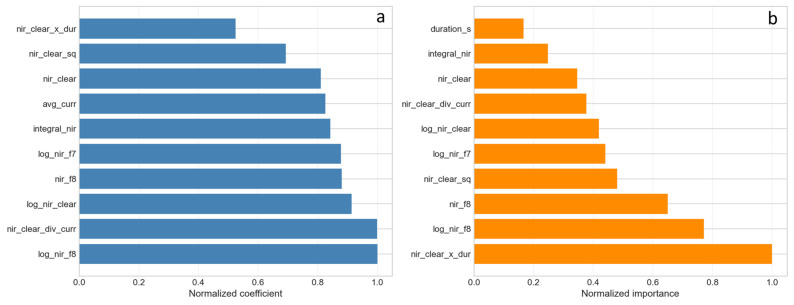
Comparison of feature importance: (**a**) ridge regression; (**b**) random forest.

Figure 7 shows a comparison of feature importance obtained by Ridge regression and Random Forest methods.

To quantify the multicollinearity among the derived features, variance inflation factors (VIFs) were calculated. The calculation was performed for the set of features that showed the highest importance in the models: log_nir_clear, log_nir_f8, nir_clear_div_curr, nir_clear_x_dur, duration_s, t_start, and avg_curr. The results are presented in Table 6.

VIF values substantially exceeding 10 indicate strong multicollinearity. This means that each of these features is almost linearly dependent on the others. Under such conditions, the coefficient estimates of ordinary linear regression become highly unstable, which led to a negative coefficient of determination (R^2^ = −0.16). The use of Ridge regression (L2 regularization) and Random Forest, both robust to multicollinearity, is therefore fully justified.

The high VIF values together with the correlation matrix (Figure 4) show that log_nir_f8 and log_nir_clear are practically interchangeable (correlation coefficient 0.95), while nir_clear_x_dur is strongly related to duration_s (r = 0.79) and to log_nir_clear (r = 0.73). To eliminate redundancy and reduce the risk of overfitting, the original feature set was reduced to three features: log_nir_clear (as the most correlated with temperature and statistically not inferior to log_nir_f8), duration_s (capturing the heating duration), and t_start (initial sample temperature). For this compact set, VIF values do not exceed 5, and it demonstrated the best metrics under validation on independent datasets (Table 7).

Thus, purposeful feature engineering—starting from the original integral channel signals, which have low importance—is a necessary step for building reliable machine learning models under limited sample size conditions.

### 3.4. Validation of the Model on an Independent Sample

To evaluate the generalization ability of the model, an independent validation was performed on samples completely excluded from the training process, taking into account different current values (50 A and 60 A). The validation was carried out as follows: the model was trained on all cycles with one current value and tested on cycles with the other current. Ridge regression was used with the features log_nir_clear, duration_s, and t_start (the set that showed the best results in the preliminary analysis). The obtained quality metrics are presented in Table 7.

Both MAE values (85.5–91.4 °C) lie within less than half the width of the temperature zone (100 °C), which allows reliable separation of the three states: underheating (<800 °C), normal (800–900 °C), and overheating (>900 °C). Comparison with LOOCV shows good agreement of the results, and the close (even slightly lower) error on the independent sample confirms that the model successfully generalizes to data with different current modes, provided that both modes were represented in the overall dataset.

**Figure 8:**
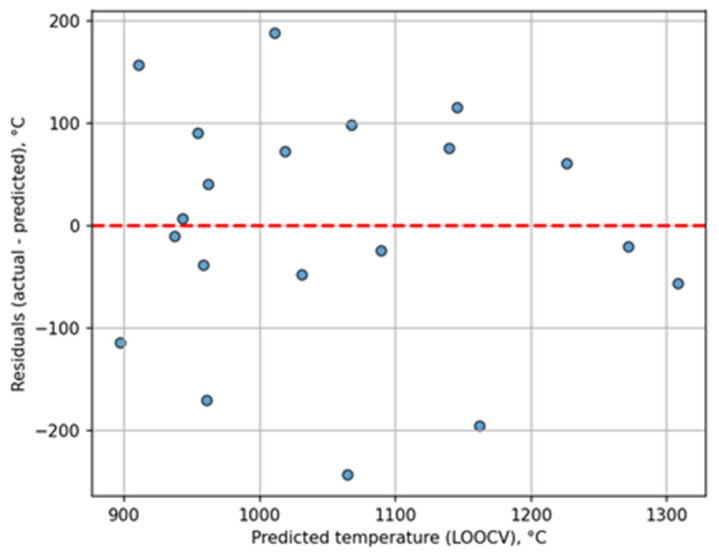
Residual plot of ridge regression based on LOOCV.

Figure 8 shows the residual plot for LOOCV. Analysis of the plot indicates that typical deviations lie in the range of 50–150 °C, with no systematic error. The normalized root mean square error (NRMSE) was 0.21 for LOOCV and 0.20 for the independent validation, corresponding to 20–21% of the temperature range (504 °C). The NRMSE was calculated over the full experimental temperature range of the sample (from 783 to 1287 °C), which is consistent with standard practice in regression model evaluation. Using an alternative temperature interval does not change the main conclusions of the work. The NRMSE values are in the range typical for thermal methods [32].

### 3.5. Metallographic Analysis

**Figure 9:**
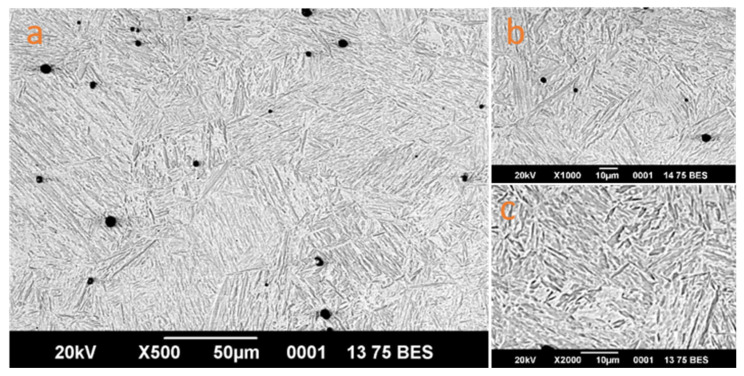
Microstructure of the cross-section of 30KhGSA steel after EPH (hardening mode) at different magnifications: (**a**) ×500, (**b**) ×1000, and (**c**) ×2000.

Metallographic examination of a 30KhGSA steel sample subjected to EPH in a mode corresponding to the hardening zone (800–900 °C according to the thermocouple readings) was carried out to verify the structural changes forming in the surface layer. Figure 9 shows an SEM image of the cross-section of the sample. The formation of fine-needle martensite is recorded in the hardened surface layer. The hardened layer is characterized by a gradient structure: surface martensite–troostite–martensite heat-affected zone, smoothly transitioning into the original ferrite–pearlite matrix. The thickness of the hardened layer was 2.5–3.0 mm, which is consistent with data from works [3,5,26].

**Figure 10:**
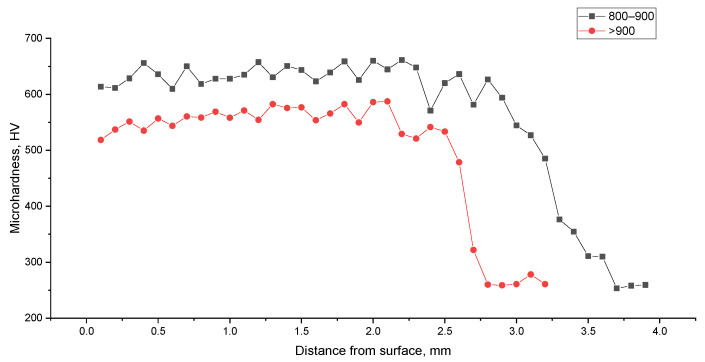
Hardness of 30KhGSA steel samples in cross-section.

Samples treated in overheating modes (>900 °C) showed coarsening of the austenite grain and signs of melting of the surface layer, which corresponds to excessive heat input. The microhardness of such samples turned out to be below optimal due to grain growth during overheating. Samples from the cold zone (<800 °C) retained the original ferrite–pearlite structure without signs of martensitic transformation or contained a small amount of troostite and martensite structures. Vickers microhardness measurements confirmed the correlation between the temperature zone and the mechanical properties of the surface layer (Figure 10 and Table 8).

## 4. Discussion

### 4.1. Physical Interpretation of NIR/Clear

Of particular interest is the log_nir_f8 feature—the logarithm of the ratio of NIR (940 nm) to channel F8 (680 nm). As shown in Table 4, this feature demonstrates a correlation of r = 0.778, which is only slightly inferior to the log_nir_clear feature. Physically, this is explained by the fact that both channels are located in the long-wave region of the spectrum, where the thermal radiation of steel is most sensitive to temperature in the range of 800–1200 °C. The ratio of two narrow bands effectively compensates for variations in absolute intensity, analogous to the principle of two-color pyrometry. The logarithmic transformation of the NIR/F8 ratio makes it possible to linearize the exponential dependence of the thermal radiation intensity on temperature, similar to the log_nir_clear feature. According to Planck’s law, the spectral density of black body radiation is proportional to

(1) $$B_{\lambda }(T)=(2hc^{2}/\lambda^{5})\;\cdot \;1/(e^{hc/(\lambda k_{B}T)}-1).$$

For two wavelengths *λ*_1_ = 940 nm (NIR) and *λ*_2_ = 680 nm (F8), the ratio of spectral intensities, taking into account the Wien approximation (when $e^{hc/(\lambda k_{B}T)}\gg 1$), can be written as follows:

(2) $$\frac{I_{680}}{I_{940}}\approx {\left(\frac{I_{680}}{I_{940}}\right)}^{5}\cdot exp\left[\frac{hc}{k_{B}T}\left(\frac{1}{\lambda_{680}}-\frac{1}{\lambda_{940}}\right)\right].$$

Taking the logarithm gives a linear dependence on the reciprocal temperature

(3) $$\ln\left(\frac{I_{680}}{I_{940}}\right)\approx 5\ln\left(\frac{\lambda_{680}}{\lambda_{940}}\right)+\frac{hc}{k_{B}T}\left(\frac{1}{\lambda_{680}}-\frac{1}{\lambda_{940}}\right).$$

It should be emphasized that the above derivations are based on Wien’s law for blackbody radiation and are valid only for the idealized case where the detector registers only the thermal radiation of the heated steel attenuated by the vapor-gas envelope. Under real EPH conditions, the plasma’s own emission–particularly intense in the visible region (channels F5–F8)–adds to this signal. Therefore, Expressions (2) and (3) describe only one component of the signal. Nevertheless, the use of the NIR/F8 spectral ratio and its logarithm helps minimize the influence of plasma emission, because plasma radiation dominates in the short-wavelength channels (F5–F8), while its contribution substantially weakens in the long-wavelength NIR channel (940 nm). The ratio of the two channels partially compensates for the plasma contribution, provided that the spectral dependence of plasma emission changes slowly over the 680–940 nm interval. It is this property that makes the empirical regression model built on spectral ratios workable, despite the fundamental physical complexity of the process.

The practical significance of this result is that two narrow spectral channels—NIR (940 nm) and F8 (680 nm)—can be sufficient for temperature estimation, without using the broadband Clear channel. This simplifies hardware implementation and potentially increases resistance to broadband interference. Thus, the log_nir_f8 feature can be considered as a linearized color temperature of the plasma emission, with the sensitivity to temperature determined by the difference in reciprocal wavelengths $\left(1/\lambda_{680}-1/\lambda_{940}\right)$.

### 4.2. Specifics of Applying Machine Learning to Small Samples

The results obtained in this work should be interpreted in the context of the general problem of applying ML in materials science and experimental sciences, where the volume of available data often does not exceed several tens of observations [1,2,17]. According to the analysis carried out in [18], the key factor limiting the accuracy of ML models at small *n* is not so much the volume of data itself, but the impossibility of using models with a high degree of freedom without the risk of overfitting. That is why in this work, the main emphasis was placed on

− Physically sound construction of features, allowing the incorporation of a priori knowledge about the process (Wien’s law, two-color pyrometry) directly into the feature space;
− The use of regularized (Ridge) and ensemble (Random Forest) models, which have proven their effectiveness in small sample conditions [21];
− The use of LOOCV to obtain an unbiased estimate of generalizing ability [22,23].

The achieved value of R^2^ = 0.466 and MAE ≈ 98 °C for the model using only the spectral data of the AS7341 should be considered not as the final accuracy of an industrial system, but as a proof-of-concept. It confirms that even on an extremely limited amount of data (*n* = 20), it is possible to identify statistically significant correlations and build a workable regression model using the correct methodology. The obtained results are consistent with known patterns; for example, the review [13,33,34,35,36] shows that for datasets of about 100–200 examples, typical errors in predicting material properties are 5–15% of the range, which is comparable to our results.

The achieved values of R^2^ = 0.483 and MAE ≈ 91 °C for the model relying solely on AS7341 spectral data confirm that statistically significant correlations can be identified and a workable regression model can be built using a correct methodology. This finding is consistent with the reviews [13,33,34,35,36], which emphasize that the proper choice of validation scheme and feature space enables statistically significant results with a small number of observations.

### 4.3. Validation Through Metallographic Analysis

An important result of this work is the independent verification of the developed monitoring system by metallographic analysis methods. The correspondence between the ML classifier classes and the actual phase composition, confirmed by scanning electron microscopy, as well as microhardness data, indicates the physical validity of the proposed approach. The three-class division (underheating—hardening—overheating) reflects the real metallurgical processes occurring in the surface layer of 30KhGSA steel during EPH [37]. The obtained microhardness values (620–680 HV for the hardening zone) correspond to typical values for hardened steel of this class [3].

## 5. Conclusions

On a controlled sample with fixed heating current (20 cycles, 50 A at 320 V and 60 A at 280–300 V), the AS7341 sensor demonstrates high informativeness for predicting the surface temperature of steel based on spectral ratios during electrolytic-plasma hardening. The main findings are as follows:

1. Channel ratios and logarithmic transformations. Absolute spectral channel values are not recommended for direct use because of their sensitivity to plasma brightness variations unrelated to temperature (e.g., changes in current). The most informative features are ratios of the NIR channel (940 nm) to Clear (broadband), F8 (680 nm) and F7 (630 nm). Switching to such ratios and their logarithmic transformations increases the Pearson correlation coefficient with the peak temperature from 0.498 (raw NIR channel) to 0.781 (log_nir_clear). This confirms that targeted feature engineering is a necessary step for reliable spectral thermometry models.
2. Informativeness of narrow channels. The feature log_nir_f8 exhibits a correlation of r = 0.778, only marginally lower than the best feature log_nir_clear. This shows that two narrow spectral channels in the long-wavelength region (940 and 680 nm) are sufficient for temperature estimation without using the broadband Clear channel. Such a solution simplifies hardware implementation and improves immunity to broadband interference.
3. Role of current as a confounder and normalization. The feature nir_clear_div_curr (NIR/Clear normalized by the cycle-averaged current) retains a high correlation (r = 0.761). Fixing the current in the controlled sample revealed the true dependence of the spectral ratios on temperature; in a real system with varying current, normalization by current or including current as a covariate in the regression is mandatory.
4. Regression models. Linear regression gave a negative R^2^ = −0.16 due to multicollinearity and nonlinearities. Applying Ridge regression (L2 regularization) improved the result: R^2^ = 0.483, MAE = 91.4 °C. Random Forest gave comparable results (R^2^ = 0.424, MAE = 98.3 °C). Independent validation on current-group splits (GroupKFold) confirmed the generalization ability: MAE = 85.5 °C, R^2^ = 0.536, NRMSE = 0.20.
5. Accounting for time scale and feature importance. For ensemble methods (Random Forest), the feature nir_clear_x_dur (product of the spectral ratio and cycle duration) is recommended because it accounts for accumulated heat input. In Ridge regression, the most important features are log_nir_f8, nir_clear_div_curr and log_nir_clear. The raw integral channel signals without transformations have low importance, confirming the necessity of purposeful feature engineering.
6. Recommended feature set and dimensionality reduction. For practical applications, 5–7 features are sufficient: log_nir_clear (or log_nir_f8), nir_clear_div_curr, nir_clear_x_dur, together with the technological parameters duration_s and t_start. Reducing the dimensionality from 62 to 5–7 lowers the risk of overfitting for a small sample (*n* = 20) and improves model interpretability.

Thus, purposefully constructing physically grounded features makes it possible to minimize the model dimensionality without loss of accuracy and is a critical step in developing virtual temperature sensors based on spectral data under limited-sample conditions. The identified regularities provide a basis for the subsequent collection of an expanded dataset (≥100 cycles) with uniform coverage of the parameter range. The proposed feature selection approach can be extended to other spectral-diagnostics tasks in plasma processes.

## Figures and Tables

**Table 1:** Parameters of the controlled sample (*n* = 20).

Parameter	Range/Values
Number of observations	20
Voltage, V	280 (7 cycles), 300 (2 cycles), 320 (11 cycles)
Heating current, A	60 A (280/300 V) и 50 A (320 V)
Cycle duration, s	7.9–15.3
Peak temperature, °C	783–1287
t_start (initial temperature), °C	35–194
Total measurement points	1371
Average points per cycle	68.5

**Table 2:** Chemical composition of 30KhGSA steel.

Element	C	Si	Mn	Ni	S	P	Cr	Cu
**Content**	0.28–0.34%	0.9–1.2%	0.8–1.1%	≤0.3%	≤0.025%	≤0.025%	0.8–1.1%	≤0.3%

**Table 3:** Correlations of AS7341 channel values with t_max_sensor.

Channel	Wavelength	r (with t_max)	*p*-Value
NIR	940 nm	+0.498	0.026
F8–F1, Clear	Broadband	<0.3	>0.05

**Table 4:** Correlations of derived features with t_max_sensor.

Feature	Formula	r	*p*-Value	Interpretation
log_nir_clear	log(NIR/Clear)	+0.781	<0.001	Very strong, statistically significant
log_nir_f8	log(NIR/F8)	+0.778	<0.001	Very strong, statistically significant
nir_clear	NIR/Clear	+0.769	<0.001	Very strong, statistically significant
nir_f8	NIR/F8	+0.766	<0.001	Very strong, statistically significant
nir_clear_div_curr	(NIR/Clear)/avg_curr	+0.761	<0.001	Very strong, statistically significant
nir_clear_sq	(NIR/Clear)^2^	+0.746	<0.001	Very strong, statistically significant
log_nir_f7	log(NIR/F7)	+0.726	<0.001	Very strong, statistically significant
nir_clear_sq_x_dur	(NIR/Clear)^2^ × duration	+0.647	0.002	Moderate, statistically significant
log_nir_clear_x_dur	log(NIR/Clear) × duration	+0.596	0.006	Moderate, statistically significant
nir_clear_x_dur	(NIR/Clear) × duration	+0.580	0.007	Moderate, statistically significant
integral_temp_model	—	+0.555	0.011	Moderate, statistically significant

**Table 5:** Correlation of log(NIR/F8) with t_max_sensor under added noise.

Noise Level	Correlation, r
0%	0.778
5%	0.763
10%	0.732
15%	0.665
20%	0.455

**Table 6:** Variance inflation factors (VIF) for the set of features.

Feature	VIF
log_nir_clear	128.8
nir_clear_div_curr	75.7
nir_clear_x_dur	113.9
duration_s	39.2
t_start	8.0
avg_curr	9.8

**Table 7:** Quality metrics of the ridge regression model under different validation approaches.

Validation Method	MAE, °C	R^2^	NRMSE
LOOCV	91.4	0.483	0.21
Independent	85.5	0.536	0.20

**Table 8:** Results of metallographic analysis and microhardness measurement.

Mode	T, °C	ML Class	Hardness HV	Layer Thickness, mm	Structure
Underheating	<800	0	280–320	—	Ferrite–pearlite
Optimal	800–900	1	620–680	3.0	Fine-needle martensite
Overheating	>900	2	540–590	2.5	Coarse-needle martensite

## Data Availability

The data are not publicly available due to privacy and technical restrictions. The data presented in this study are available on request from the corresponding author.
